# Supplementary figures and images for: Incidental temporal binding in rats: A novel behavioral task
Source: PLoS One. 2023 Jun 22;18(6):e0274437. doi: 10.1371/journal.pone.0274437 (PMC10286974; doi:10.1371/journal.pone.0274437)

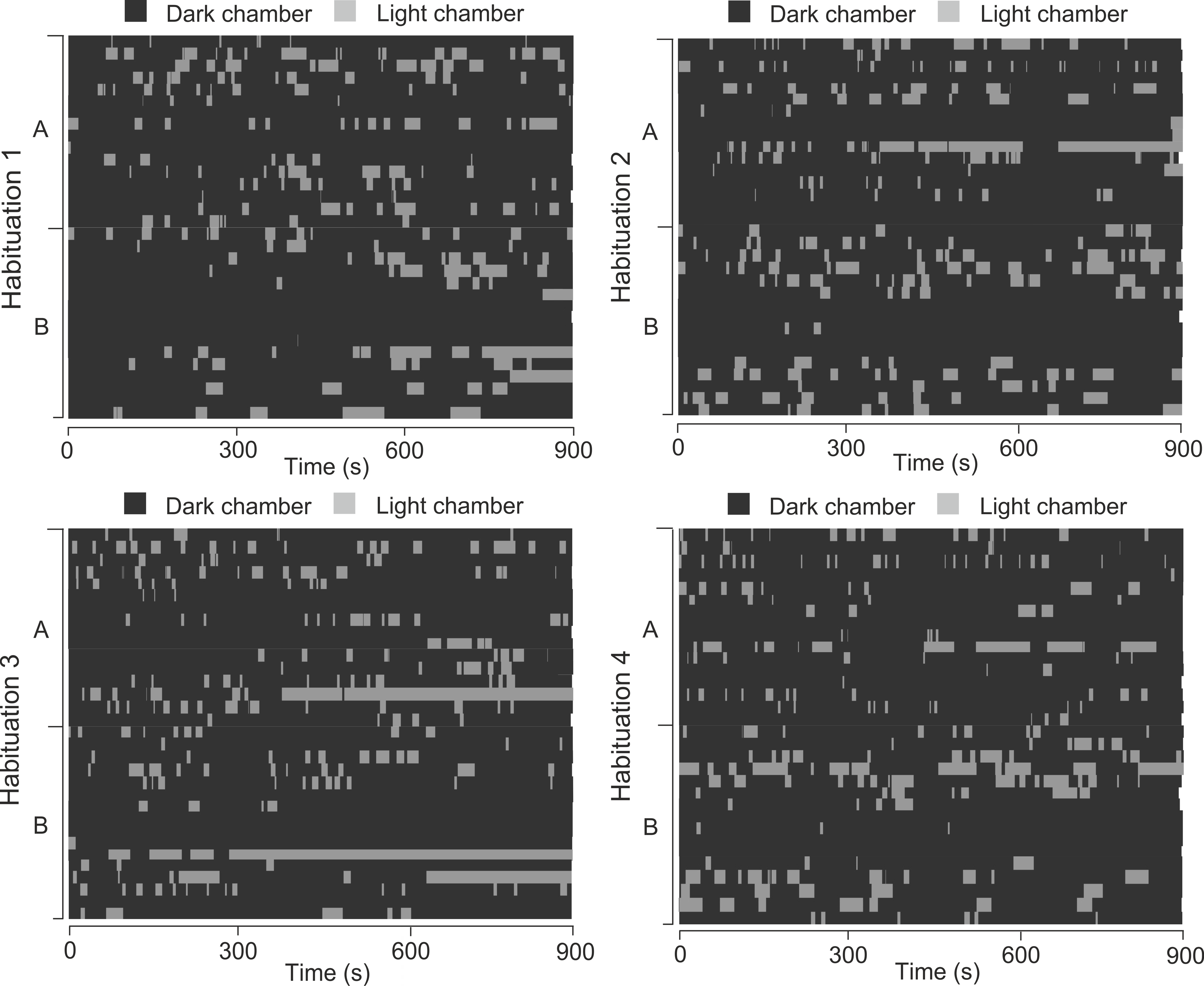

Supplement: S1 Fig — Each horizontal line corresponds to one animal. Black represents a stay in the dark chamber; gray represents a stay in the light chamber. Symbols "A" and "B" on the vertical axis correspond to the environmental context of the rat’s habituation. Context A was oval-shaped and cleaned with an alcohol-based wash, while context B was rectangular-shaped and cleaned with a vinegar-based wash. (TIF) [file pone.0274437.s001.tif]

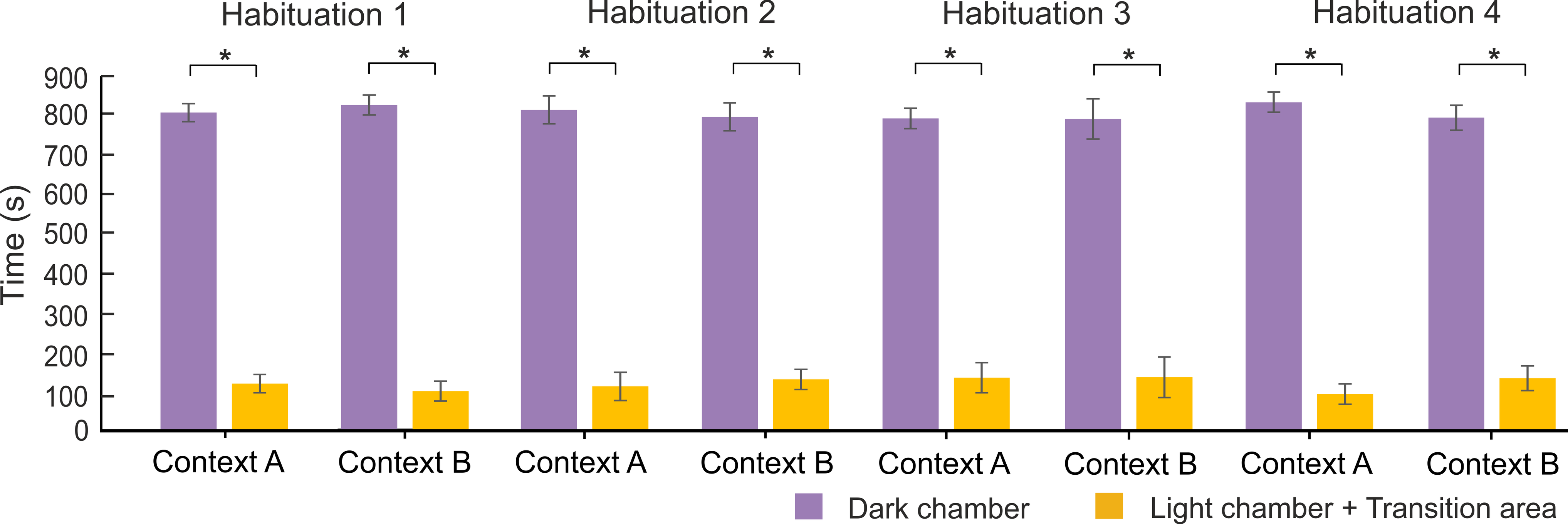

Supplement: S2 Fig — Each rat (N = 32) received two habituation sessions in each context in an alternating manner. Starting context was chosen randomly for each rat. Rats preferred the dark chamber and spent very little time in the light chamber in both contexts across habituation sessions. The difference in the time spent in the dark chamber was significantly higher than the time spent in the light chamber in each context and during all five habituation sessions (Mann-Whitney test: Hab 1 context A: U = 0.000, p = 0.000; Hab 1 context B: U = 0.000, p = 0.000; Hab 2 context A: U = 1.000, p = 0.000, Hab 2 context B: U = 0.000, p = 0.000; Hab 3 context A: U = 1.000, p = 0.000, Hab 3 context B: U = 6.000, p = 0.000; Hab 4 context A: U = 1.000, p = 0.000, Hab 4 context B: U = 0.000, p = 0.000. Error bars indicate SEM, and * indicates p <0.05. (TIF) [file pone.0274437.s002.tif]

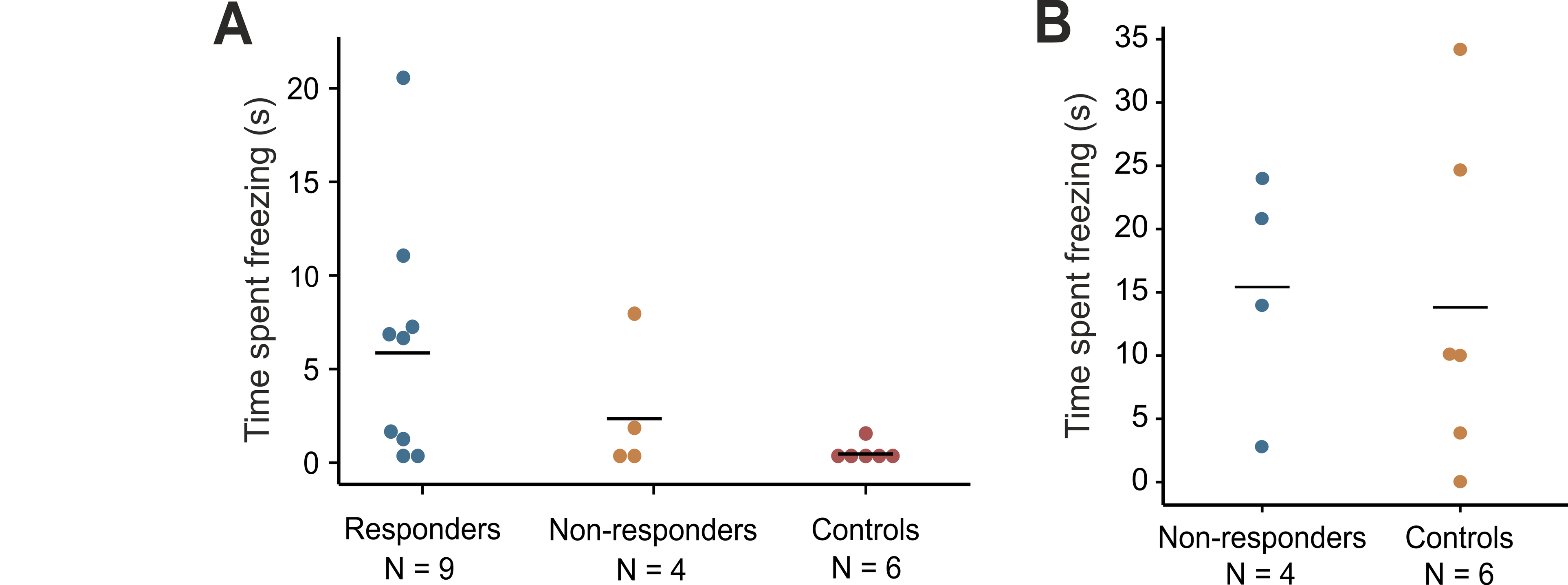

Supplement: S3 Fig — Freezing was assessed manually from video recordings by a blinded experimenter. As a freezing, we considered any lack of movement except for breathing. (A) Time spent freezing (s) during the first minute of the recall session. There was no significant difference in time spent freezing between ’responders,’ ’non-responders,’ and control rats (Kruskal-Wallis test: H = 6.436, p = 0.092). (B) Time spent freezing (s) during one minute after CS presentation. We found no significant difference in time spent freezing between ’non-responders’ and control rats (Mann-Whitney test: U = 11.00, p = 0.831). This suggests that ’non-responders’ did not recall the CS-2s-US association. (TIF) [file pone.0274437.s003.tif]
