# Supplementary material for: Incidental temporal binding in rats: A novel behavioral task
Source: PLoS One. 2023 Jun 22;18(6):e0274437. doi: 10.1371/journal.pone.0274437 (PMC10286974; doi:10.1371/journal.pone.0274437)
Supplement: Attachment — Submitted filename: Response to Reviewers.docx [file pone.0274437.s004.docx]

Responses to reviewers

Reviewer #1:

**The experiments reported in this manuscript were directed at rodent task development in the learning and memory domain. Specifically, the authors were interested in developing a behavioural task to assess episodic memory in the rat with an emphasis on a task in which the experience occurs only once, an important component of episodic memory.**

**In my opinion, the work is well-motivated, and the authors have developed an interesting task with some strong design features. The results are interesting and clearly presented and analyzed.**

**I do have a couple of significant issues with the experiments and manuscript.**

**First, I think it is a bit of a stretch to suggest that this task is tapping into the temporal component of episodic memory in which the organism must learn about when the event occurred. This paradigm simply utilizes a standard Pavlovian conditioning procedure with a very short trace (2 seconds). I don’t see any evidence presented that they learned this temporal information, just that they learned the association between the CS and US.**

**Unless I am missing something here this would change some of the content of the introduction and discussion.**

*Thank you for the positive feedback and the comment. Indeed, we cannot judge if the organism ‘learns* ***when*** *the event occurred'. We changed parts of the introduction and discussion and hope they are not misleading anymore. Changes related to this issue are highlighted in red in the attached manuscript file. We added a sentence that temporal binding is assessed using trace-conditioning tasks (in red; lines 31-32, line 415). We changed references to ‘knowledge acquired” to ‘formation’ of the association (****lines 245-246, line 356****) We hope we responded to this question appropriately.*

**Second, the fact that only 59% of the subjects learned the task is a positive and a negative. It is the former for a variety of reasons including the ability to understand individual differences in one-trial learning. It is a negative, in my view because it is probably the case if the whole group of subjects conditioning results was graphed and analyzed together there would be no effect. One potentially helpful approach would be to measure heart rate conditioning simultaneously. What you might find is that most of the subjects did learn the CS-US association but not all of them showed it in their avoidance behaviour. I understand the freezing behaviour presented suggests otherwise but conditioning measured using freezing might require multiple trials and/or several reinforcers to emerge.**

*Thank you for the comment and, yes, we agree. We tried measuring the heart rate in the past during the OTTER learning and recall but a signal from our wireless telemetry probes was canceled out by experiments being conducted in an electrified box. In the manuscript, we erased the part where we claim that responders and non-responders can be confidently classified (****lines 425-427).***

*Moreover (this overlaps with the response to the other reviewer’s comment), the percentage of responders in the OTTER corresponds to the number of humans that successfully recall the memory following incidental learning in an experimental setting (in humans the successful recall occurred little more often, in about 70% of cases). For this reason, we believe that (1) there is a likelihood that there will be rats without the memory of the CS-2s-US in the OTTER and (2) that the OTTER is an ecologically valid task. We added the information about human tasks to the text (in purple; lines 417-418):*

*“It is also ecologically valid in relation to human incidental memory: recall in incidental memory tasks was reported to be 35%-90%, depending on the task [41–43].”*

*Again, thank you for the comment, and, yes, it would be great if we could have heart rate data.*

**Third, one of the main issues the authors raise about this field of inquiry is that many episodic paradigms using rodents require shaping, training, reinforcement, etc. The authors claim that their paradigm avoids these issues. Clearly, this is true concerning some of these design features, but I would consider the repeated pre-exposure phase as training that is important to show this one-trial learning.**

*Thank you for the comment. We did not consider habituation to the apparatus to be pretraining. However, your point is a very good one and we added your suggestion to the text and explained why we do not agree (in blue; lines 331-333):*

*“It could be argued that repeated habituation is also 'pre-training'. However, repeated habituation is not pre-training in the sense that it creates the expectancy for the contingency; habituation simply reduces the incidence of variable behaviors"*

*But, we also narrowed the term pre-training in the introduction (in blue; line 36):*

*“We believe that the acquisition of episodic~~-like~~ memory should not require conditioning or pre-training to to-be-learned information; moreover, evidence suggests that mechanisms of incidental memory acquisition might differ from acquisition with intent”*

**Finally, I think a stronger demonstration of scholarship is needed. There have been a variety of tasks designed to assay episodic memory in rodents, birds, and monkeys and this work should be integrated.**

*Thank you for the suggestion. We tried our best and added a section to the discussion of the manuscript. In short, we discuss how OTTER is related to avoidance learning and other behavioral tasks. As the section is lengthy, please see the manuscript (in green; lines 253-319). Hopefully, you will find the section to your liking.*

**Reviewer #2**:

**The authors describe a one-trial avoidance learning task that incorporates a brief trace (2 sec) between the presentation conditioned stimulus and unconditioned stimulus. The authors argue that because of this trace, the single learning trial, and that the memory test is performed in a different context that the memory is episodic.**

**The behavioural task is somewhat interesting, but the argument that it demonstrates episodic memory is weak and not compelling. Rather than demonstrating that the memory meets the standard features of episodic memory (what, where, and when), they limit their argument to what they term incidental learning. However, their task does not offer much novelty or more than other tasks:**

**one-trial inhibitory avoidance (Parent, M. B., Avila, E., & McGaugh, J. L. (1995). Footshock facilitates the expression of aversively motivated memory in rats given post-training amygdala basolateral complex lesions. Brain Res, 676(2), 235-244.)**

**One-trial socially transmitted food preference (Winocur, G., McDonald, R. M., & Moscovitch, M. (2001). Anterograde and retrograde amnesia in rats with large hippocampal lesions. Hippocampus, 11(1), 18-26.)**

**The authors must have a proof of concept demonstrating that the task involves episodic memory. They should also demonstrate that lesions that impair episodic memory in humans also impair memory in this task.**

*Thank you for the comment. During the writing of the manuscript, we were cautious not to over-interpret the OTTER task as an episodic-like memory task. For that reason we used phrases such as ‘the OTTER is relevant to episodic memory’ and that it ‘models several aspects of the episodic memory’ (samples highlighted in yellow). A behavioral task does not have to model all aspects of episodic memory to be useful in learning about episodic memory. This is why ‘episodic memory’ is often mentioned in the text. However, we scrutinized the text and found some statements that could be interpreted as presenting the OTTER to be an episodic memory task. We added an explicit statement that we do not consider the OTTER to test true episodic memory (lines 374-375):*

*“Although we do not consider OTTER to be a true episodic-like memory task according to present criteria, we consider OTTER to be highly relevant to episodic memory”*

*Thank you for pointing out these issues.*

*The two other tasks that you mentioned are similar in several aspects but different in others. Parent et al., 1995 harness light and dark chambers in a similar manner as the OTTER, but it does not include the trace-conditioning aspect. We did not know this article before and we are happy that we are on a right track. Winocour et al., 2001 involve one-trial incidental learning, albeit social, but this task also does not involve the association of two events. Moreover, the behavior is likely evolutionary wired. The task is very interesting.*

**The authors should report on longer trace intervals than 2 seconds. How does performance relate to the duration of the trace.**

*Thank you for the suggestion. We did not test longer intervals, as we wanted to have the highest yield of the learning rats possible. We presume that the performance will deteriorate with the increase of the trace interval, as CS-2s-US is presented only once in the OTTER task. Moreover, it is possible that with the increasing length of trace the learning shifts towards dissociation between CS and US (doi:* [*10.1016/j.beproc.2013.08.016*](https://doi.org/10.1016%2Fj.beproc.2013.08.016)*), which we were not aiming for.*

**The task is not very pragmatic as only 50% of the rats show the desired learning and avoidance behaviour.**

*That is true. However, 50% is sufficient if differences in neurobiology between responders and non-responders are the aim of the research. From our perspective, the non-responders are the best control group for the responders. Moreover, this level of performance is ecologically valid in relation to human episodic memory. Based on your comment we added a line in a discussion (highlighted in purple, lines 417-418):*

*“It is also ecologically valid in relation to human incidental memory: recall in incidental memory tasks was reported to be 35%-90%, depending on the task [41–43].”*

**In sum, this is a methods paper and insufficient for publication. It would be best if accompanied with an experimental manipulation (e.g., lesion or inactivation).**

*We appreciate your opinion.*
