# Supplementary material for: Incidental temporal binding in rats: A novel behavioral task
Source: PLoS One. 2023 Jun 22;18(6):e0274437. doi: 10.1371/journal.pone.0274437 (PMC10286974; doi:10.1371/journal.pone.0274437)
Supplement: Attachment — Submitted filename: Response to Reviewers.docx [file pone.0274437.s005.docx]

**Response to the reviewers**

27^th^ Feb 2023

We express our gratitude to the reviewers for providing us with constructive feedback. We concur with their assessment that the task primarily serves as a test of classical conditioning rather than episodic memory. To acknowledge this fact, we have revised the introduction section of the manuscript and incorporated several modifications to the discussion section. Yet we believe that the task, being incidental and limited to a single trial, does represent some fundamental components essential for establishing episodic-like memories. Nevertheless, the nature of the task does not allow us to conclude that it is a definitive episodic memory task.

**Reviewer #1**: The authors have responded to most of my concerns appropriately and they have softened the claims about this paradigm as a measure of episodic memory. However, I am still concerned about whether this is a measure of episodic memory as this increases the interest the paradigm might garner in the field.

As it stands I do not see convincing evidence of this. Certainly they have a subset of animals that show incidental learning which is interesting but I am not sure it is publishable as the experimental design stands now (longer traces would be helpful).

**Response to Reviewer 1**: Thank you for your comment. We would like to clarify that our aim was to highlight that the OTTER task assesses several aspects that are relevant to episodic memory, but it is not designed as a specific test of episodic memory. There are many different tasks that explicitly target only some aspects of episodic memory, and they are still considered valuable in this line of research. Therefore, we believe that the OTTER task has a place among these tasks and can contribute to our understanding of the underlying mechanisms of episodic-like memory formation. We appreciate your feedback and hope that this clarification will address your concerns.

**Reviewer 1**: One approach might be to frame the paradigm within the idea that maybe all new one-trial memories are initially episodic and your incidental task captures this phenomenon (see Heterarchic reinstatement of long-term memory: A concept on hippocampal amnesia in rodent memory research. Lee JQ, Zelinski EL, McDonald RJ, Sutherland RJ. Neurosci Biobehav Rev. 2016 Dec;71:154-166.)

**Response to Reviewer 1**: Thank you for your helpful suggestion. We utilized the arguments presented in this publication to support the relevance of the OTTER task to the field of episodic memory research. Lines 37-40: *“Finally, it has been proposed that one-trial memories are initially encoded as episodic, and that different memory systems might differ in their learning rates [12]. Our task featuring one-trial learning might prove helpful in testing this hypothesis.”*

**Reviewer #2**: Unfortunately, the authors have not addressed my concerns. The entire manuscript revolves around episodic memory and both I and the other reviewer explicitly stated that there is no compelling evidence that this task relates to episodic memory. As indicated by the other reviewer, this is Pavlovian conditioning task.

The authors state "relevance" to episodic memory:

"A behavioral task does not have to model all aspects of episodic memory to be

useful in learning about episodic memory. This is why ‘episodic memory’ is often

mentioned in the text. However, we scrutinized the text and found some statements

that could be interpreted as presenting the OTTER to be an episodic memory task. We added an explicit statement that we do not consider the OTTER to test true episodic memory (lines 374-375)"

If it is not modeling episodic memory, then why is the entire manuscript developed around that topic?

Again, I think this is better suited as method paper for a new pavlovian task and not one within the episodic memory context.

**Response to Reviewer 2**: We apologize that our revisions did not meet your expectations, but we respectfully disagree with your assessment.

Following the feedback received, we have made revisions to the paper to clarify that the OTTER task is not intended to model episodic memory, nor is it designed as an episodic-like memory task, as defined by the widely accepted "what-when-where" criteria. Despite this, we believe that the task remains a valuable and relevant tool for researchers in the field of animal research who seek to investigate the nature and dysfunction of human episodic memory.

We find the OTTER task relevant to episodic memory for several reasons, despite it not being an episodic-like memory task:

- Natural human episodic memory in the everyday ecological context has a ‘one-trial’ character: the events that are later recollected as an episodic memory occur only once (it would be logically incoherent to say that exactly the same event occurred twice, since the time would be different; thus another, albeit similar, event has occurred later),
- The human episodic memory is also incidental: at least some (and possibly most) of the episodic memories that a person acquires were not committed to memory intentionally, the person did not prepare for ‘a study session’ with the intent to remember the episode and all of the elements that are later recollected,
- The OTTER task is both one-trial and incidental in character.

These important aspects of human episodic memory are severely understudied in animal paradigms that aim to elucidate human episodic memory. In our opinion, the lack of research on these aspects of episodic memory is due to the lack of suitable behavioral memory-binding tasks with one-trial and incidental characteristics.

Another way to express why we consider the OTTER task to be relevant to episodic memory and of potential interest to the field even though it does not model episodic memory is illustrated by the following analogy:
For example, an animal can never (by definition) be said to suffer from schizophrenia (the diagnostic criteria require the presence of hallucinations and delusions - both of which are personal experiences that can not be assessed in animals by a clinical interview). A similar situation applies to episodic memory, defined as a memory of subjective, personally experienced event.

Thus, people model schizophrenia in animals so that animals display schizophrenia-like behavior. The same goes for episodic-like behavior.

In schizophrenia, the reduction of the pre-pulse inhibition of the startle response (PPI) is well-known phenomenon that is present in patients and has been researched for decades. However, it is not among the diagnostic criteria (i.e. the definition of schizophrenia). It is questionable if modeling only this aspect in animals constitutes a schizophrenia model (at best, it would model symptom that is not even necessary for the patient to be diagnosed with schizophrenia). And studying healthy, undisrupted PPI definitely does not model schizophrenia. So if a researcher were to investigate the physiologically normal processes underlying PPI in a completely healthy animal, they would not model schizophrenia and elicit no schizophrenia-like behavior. But it would still be highly relevant to schizophrenia and of considerable interest to the field as clarifying the underlying processes of PPI could aid in deciphering changes occurring in schizophrenia patients. And it would be correct and even desirable for the field of schizophrenia research should the author write the paper with an eye on the topic of schizophrenia. And even more so, in a hypothetical case, if the field was not paying much attention to the PPI physiology and dysfunction due to the lack of suitable behavioral paradigms for animals (and PPI not being part of the diagnostic definition of schizophrenia).

As reasoned above, the human episodic memory likely has one-trial and incidental character, but episodic memory is not defined based on these characteristics. From the standpoint of prevailing definition (human episodic or animal episodic-like memory), the characteristics of being incidental and one-trial are non-essential, accidental - same as the reduction of PPI in schizophrenia patients is unnecessary for the diagnosis, yet still highly relevant for the field of schizophrenia research.

We hope that we showed clearly why we consider the OTTER task to be relevant to episodic memory even though it is not a what-when-where episodic memory-like task. We really appreciate your comment as it prompted us to think very critically about arguments supporting OTTER as a episodic memory task. We have reworked the paper based on the both reviewers’ feedback and now clearly state in the paper that the OTTER is not an episodic memory task. However, we still consider the task to be relevant to episodic memory and of potential interest to the field.
