# Supplementary material for: Incidental temporal binding in rats: A novel behavioral task
Source: PLoS One. 2023 Jun 22;18(6):e0274437. doi: 10.1371/journal.pone.0274437 (PMC10286974; doi:10.1371/journal.pone.0274437)
Supplement: Attachment — Submitted filename: response to reviewers.docx [file pone.0274437.s006.docx]

**Responses to Reviewer #2**

15.5.2023

**Reviewer #2**: This is an interesting task and could be of some use. Unfortunately, I still have issues related to the contextualization around episodic memory. I still do not understand how this task is different than other one-trial tasks.

**Radostova et al.**: We greatly appreciate your partial approval of the manuscript. We would like to clarify that the OTTER task is not claimed to be entirely distinct but expands the range of existing one-trial tasks. What we find particularly original about the OTTER task is its utilization of two types of motivation to influence animal behavior.

**Reviewer #2**: The incidental argument, in my view, is poorly presented. The authors, in the first paragraph of the introduction, define incidental as "memory that can be tested when subjects are unaware that they will be tested on recall". When would rats be aware that they will be tested for memory? Basically, the authors' operational definition for rodent memory is inadequate.

**Radostova et al.**: We apologize for not fully explaining this manuscript part. We added a following section: “*To ensure that memory encoding is incidental, the memory test should be unexpected when the to-be-remembered event occurs [Zentall 2013]. Repeated test trials can lead animals to anticipate future memory testing. This repetition helps animals learn the task rules and identify relevant information, making them more motivated to encode the information intentionally”.*

Hopefully, this clarifies the issue.

**Reviewer #2**: Similarly, the first sentence of the second paragraph emphasizes the need to study time gaps to better understand episodic memory, which again I do not find supported by the literature on episodic memory. The importance, within the episodic memory literature, is the time stamp between events (the sequence of two different events). This task assesses a CS-US association and not a time stamp or sequence of events. Moreover, the authors do not study time gaps in this manuscript as they only examined one interval between the CS and US.

**Radostova et al.**: We changed the term ‘episodic memories’ to ‘complex memories.’ We think this minor change resolves your concerns about the paragraph.

**Reviewer #2**: I stand firm that the introduction should revolve around a new one-trial conditioning task. The episodic memory component should be reduced.

**Radostova et al.**: We did as you requested: we erased all mentions of the ‘episodic’ memory (but one, requested by the other reviewer) from the introduction paragraph. Thank you for this suggestion, as we think addressing all complex memories is an added value.

**Reviewer #2**: The discussion is improved, but the "declarative" argument in the discussion should be revised as it is much too strong and overstated for this task. Declarative memory implies describing facts and events (use of language). The OTTER task does not provide the richness of detail associated with declaration.

**Radostova et al.**: We appreciate your perspective on the matter. We have replaced the term "declaring" with "showing" and the phrase "declarative behavior" with "response." Thank you for your valuable comment. We believe the paragraph now conveys the intended meaning more appropriately.

**Reviewer #2**: Further the authors should briefly discuss the view that rodents may not have episodic memory (see Zenthal, 2013).

**Radostova et al.**: Thank you for your feedback. Considering your suggestion, we have removed all statements related to episodic memory as advised. Therefore, there is no need further to discuss the presence of episodic memory in rodents. However, we did cite Zentall 2013 in the introduction when discussing ‘unexpected question’ tasks.

**Reviewer #2**: I strongly recommend that the authors read and come to cite these two papers:

- Pause, B. M., Zlomuzica, A., Kinugawa, K., Mariani, J., Pietrowsky, R., & Dere, E. (2013). Perspectives on episodic-like and episodic memory. Front Behav Neurosci, 7, 33. doi: 10.3389/fnbeh.2013.00033
- Zentall, T. R. (2013). Animals Represent the past and the Future. Evolutionary Psychology, 11(3), 147470491301100307. doi: 10.1177/147470491301100307

**Radostova et al.**: We appreciate your valuable recommendations and find the suggested reads highly interesting. However, since we have significantly reduced references to episodic memory in our discussion, citing these sources is no longer relevant.
